# Group B streptococcal colonization in mothers and infants in western China: prevalences and risk factors

**DOI:** 10.1186/s12879-018-3216-4

**Published:** 2018-07-03

**Authors:** Jichang Chen, Jinjian Fu, Wei Du, Xin Liu, Chokechai Rongkavilit, Xuemei Huang, Yubi Wu, Yuanliu Wang, Eric McGrath

**Affiliations:** 1 Department of Neonatology, Liuzhou Maternity and Child Health Care Hospital, Liuzhou, 545001 China; 2 Department of Laboratory, Liuzhou Maternity and Child Healthcare Hospital, Liuzhou, 545001 China; 3 Children’s Hospital of Michigan, Detroit, MI USA; 4 Department of Pediatrics, Wayne State University School of Medicine, Detroit, MI USA; 5 Department of Pediatrics, Liuzhou Maternity and Child Healthcare Hospital, Liuzhou, 545001 China; 6 Department of Infectious Diseases, Valley Children’s Hospital, Madera, CA USA; 7 Department of Obstetrics and Gynecology, Liuzhou Maternity and Child Healthcare Hospital, Liuzhou, 545001 China; 8 Division of Infectious Diseases, Children’s Hospital of Michigan, 3901 Beaubien Boulevard, Detroit, MI 48201 USA

**Keywords:** Group B streptococcus, GBS colonization, Early onset disease (EOD), Mother-infant pairs

## Abstract

**Background:**

The epidemiology of maternal and infant Group B streptococcus (GBS) colonization is poorly understood in China. The aim of this study is to explore the prevalence and risk factors associated with maternal and infant GBS colonization in Western China.

**Methods:**

From January 2017 to June 2017, a prospective study was conducted to estimate the maternal and infant GBS colonization rate by maternal rectovaginal and infant nasopharynx, ear canal and umbilical swab culture. Patient demographics, clinical characteristics and outcomes were collected. Chi-square and logistic regression analyses were used to examine the risk factors associated with GBS colonization of mothers and infants.

**Results:**

The GBS colonization rate in mothers and infants was 6.1 and 0.7%, respectively. The vertical transmission rate was 7.6%. The early onset GBS infection rate was 0.58 per 1000 live births and mortality was 0.29 per 1000 live births. Age younger than 40 years (*p* = 0.040) and minority ethnic status (*p* = 0.049) were associated with higher GBS colonization rate in pregnant women. Positive GBS status in the mother prior to delivery (*p < 0.001*) as well as longer duration of membrane rupture (≥12 h) (*p* < 0.001) and longer labor (≥4 h) (*p < 0.001*) were all significant risk factors for GBS colonization in infants. Compared to infants without GBS colonization, infants colonized with GBS were more likely to have had a temperature of ≥38 °C (*p* < 0.001), developed early onset infection (EOD) (*p* < 0.001), and been prescribed antibiotics (*p* < 0.001). Furthermore, infants with GBS were more likely to have been admitted to neonatal intensive unit (NICU) (*p* < 0.001) with a longer hospital length of stay (LOS) (*p* < 0.001).

**Conclusions:**

Maternal GBS colonization, longer duration of membrane rupture and labor were all major risk factors associated with GBS colonization in Chinese infants. Infant GBS colonization was associated with increased risk of EOD and NICU admission as well as longer LOS.

## Background

*Streptococcus agalactiae*, known as Group B streptococcus (GBS), is a common colonizing pathogen in pregnant women that can cause invasive infections during peripartum period [1]. The Vaginal and rectal tract are considered major reservoirs of GBS [2]. Mucosa surfaces such as oral, nasopharyngeal, vaginal and anal mucosa and skin of newborns at birth support the colonization of GBS [3]. It was reported that the risk of colonization of GBS was much higher in infants who were born to GBS positive mothers, and the likelihood was even higher if the mother was heavily colonized (growth of GBS with a density between 3^+^ to 4^+^) [4, 5]. GBS can easily be transmitted vertically from mothers to newborns in the peripartum period, especially during vaginal delivery [6]. GBS has been the principal cause of septicemia, meningitis and pneumonia for early and late onset diseases in newborns and was attributed to infant mortality in many parts of the world [4]. Little is known about the burden of perinatal GBS colonization and related infections in China. Specifically, the prevalence of maternal and infant GBS colonization and the risk factors for vertical transmission of GBS from mothers to infants have not been reported in western China. Since there is no screening for GBS or intrapartum administration of antibiotics (IAP) protocol was in place in China for prevention of GBS disease at the time of this study, the aims of this study were to determine the prevalence of and risk factors for maternal and infant GBS colonization at Liuzhou Maternity and Child Healthcare Hospital (LMCHH), one of the largest maternal and child health care hospitals in Guangxi in western China, in order to inform future prevention efforts and interventions.

## Methods

### Study design

This was a prospective mother-infant pair study conducted between January 2017 and June 2017 at LMCHH in Liuzhou, Guanxi Province in western China. This study recruited mother-infant pairs from LMCHH during the study period. The study included pregnant women (≥35 weeks of gestation) visiting the antenatal clinic for routine prenatal care and those who were approached at time of delivery. If no sample was collected during these routine prenatal visits, then they would be swabbed at the time of delivery.

The exclusion criteria included: (1) all twins or multiple births (2) in any case where GBS culture results were not obtainable; and (3) the screened pregnant woman delivered outside LMCHH. An oral consent was obtained from participating women and the study was approved by the Institutional Review Board at LMCHH.

GBS screening was per protocol as part of this study’s procedures. No routine screening for GBS or intrapartum administration of antibiotics (IAP) protocol were in place in China at the time of this study. If pregnant women had additional clinical risk factors such as fever and/or preterm birth, then antibiotics may have been given at the discretion of the treating clinician, but there was no formal IAP protocol which was followed.

## Data collection

Women and newborns were followed during the entire delivery hospitalization in LMCHH. The microbiological, clinical records and hospital databases were reviewed and recorded at the end follow-up for each pair of participants. Demographics, microbiological and clinical records from hospital databases were reviewed and extracted by study investigators in LMCHH. Data elements included in this study were: maternal age, previous obstetric history, gestational age, parity, mode of delivery, duration of membrane rupture (in hours), labor duration (in hours), antibiotic use of mother and newborn, newborn gender, birth weight, neonatal fever, newborn length of stay (LOS), occurrence of early onset diseases (EOD), and neonatal intensive care unit (NICU) admission.

### Definition

***Fever*** [7]: A temperature of ≥38 °C in newborns was considered as the threshold for fever.

***Early Onset Disease (EOD)*** [5]: A diagnosis by way of isolation of bacteria from sterile sites such as blood or CSF within 7 days after birth.

***Prolonged rupture of membranes*** [8]: Membrane rupture of ≥12 h was considered as the threshold for prolonged rupture of membranes.

***Prolonged labor***: Since there was no standard definition of prolonged labor, labor duration of ≥4 h was used as the cut-off value of prolonged duration of labor for this study.

Minority ethnic group: There are 56 different ethnic groups in China. Han and Zhuang are the two major ethnic groups in Guangxi, which consist of about 90% of the population in Guangxi. The minority ethnic group is defined as any other ethnic groups except Han and Zhuang.

### Specimen collection and microbiologic methods

Vaginal and rectal swabs as two different swabs were collected from pregnant women for bacterial cultures at 35–37 weeks of gestation by their obstetrical/gynecological physician. If the bacterial culture was not done at around 35–37 weeks, vaginal and rectal swabs were collected at the onset of labor. One sample swab from newborns was collected by the treating perinatologist in the order of nasopharynx, then ear canal and final umbilical cord. Swabs were collected from newborns immediately after birth (< one hour).

Specimens were collected using sterile swabs then placed in Stuart transport medium and sent to laboratory within 2 h of obtaining the samples. The swabs were incubated in a selective enrichment broth medium (Todd Hewitt broth) (OXOID, England) containing nalidixic acid (15 mg/L) and gentamicin (8 mg/L) at 37 °C for 18–24 h. The broths were sub-cultured in Columbia 5% sheep blood agar (Autobio, China) and incubated at 37 °C in 5% CO_2_ for 18 to 24 h. Colonies were identified presumptively by colony morphology, Gram stain, catalase reaction, hemolytic activity on sheep blood agar plates, Hippurate and CAMP test. The suspected colonies were further tested with Gram positive bacteria identification card using VITEK 2 compact automatic microbial analysis system (Biomérieux, Marcyl’ Etoile, France) to confirm GBS isolates.

### Statistical analysis

SAS version 9.4 (Cary, NC, USA) was used to perform the statistical analysis. Continuous variables were analyzed using 2-sample *t*-tests. Categorical variables were analyzed and compared by chi-square or Fisher’s exact tests. Odds ratios (ORs) and their 95% confidence intervals (CIs) were calculated to assess the relative risk associated with maternal and infant GBS colonization. Multivariate logistic regression in conjunction with Stepwise backward elimination model selection method was performed to evaluate the relative strength of potential risk factors for GBS colonization in mothers and infants separately. A *p*-value less than 0.05 was considered indicative of statistical significance.

## Results

### Enrollment

**Fig. 1:**
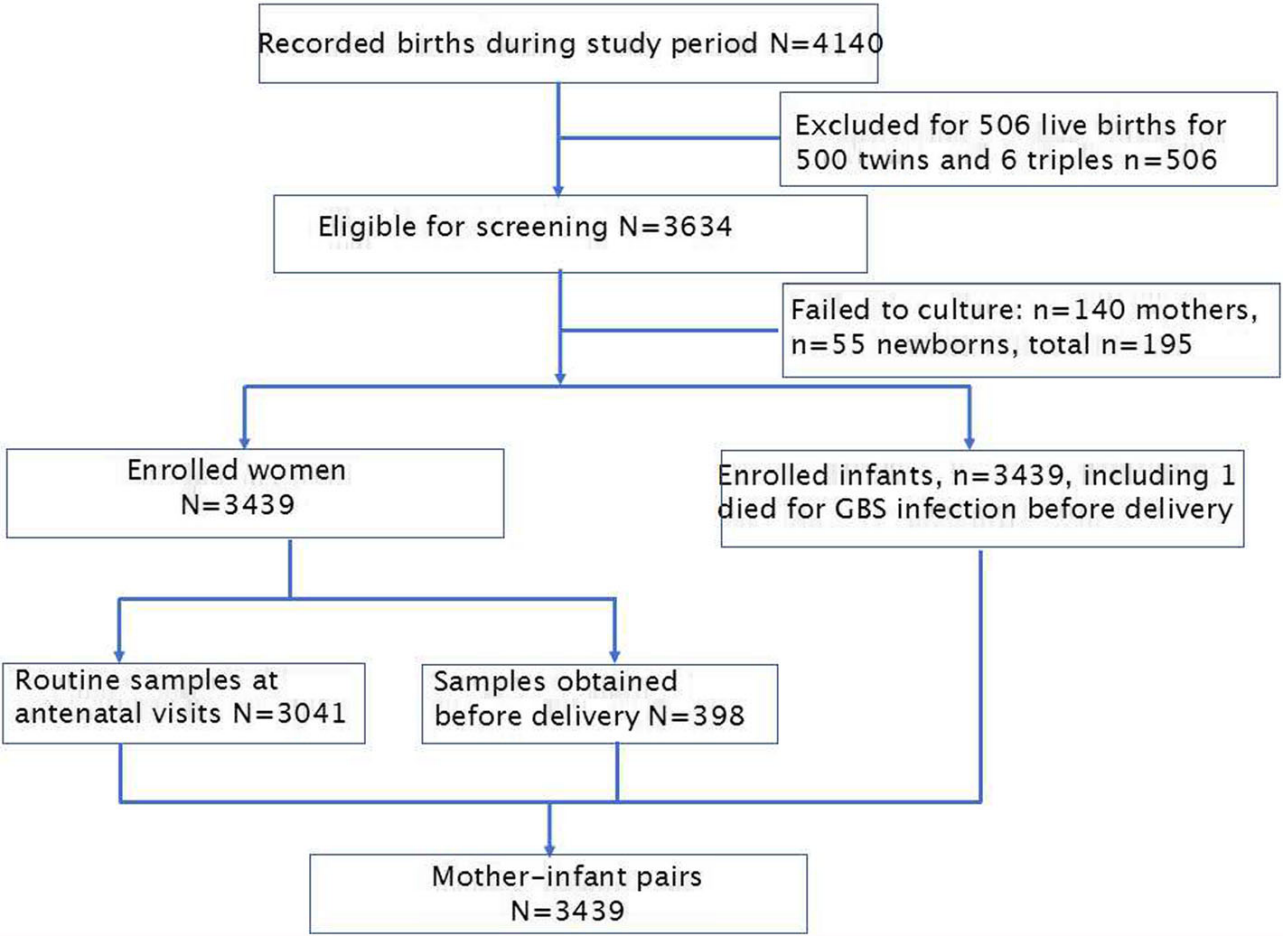
Enrollment flow chart

Data from 4140 births were collected and recorded during the study period (see Fig. 1). Seven hundred and one were excluded. This left a total of 3439 births that were eligible to be included in the study.

### Maternal data

Among the 3439 specimens from mothers, 2841 were obtained during their routine prenatal visits while 598 specimens were collected at the onset of labor. Among 3439 women, GBS was isolated in 210, and the overall colonization rate was 6.1%. Table 1 examined the characteristics of mothers as potential risk factors for GBS colonization. A multivariate analysis has found that age younger than 40 years (*P* = 0.040) as well as being in the minority ethnic group (*p* = 0.049) were significant risk factors for GBS colonization (Table 2).

**Table 1:** Association between risk factors and GBS colonization among pregnant women

Variables	GBS carriers (*n* = 210) (*n*,%)	GBS negative (*n* = 3229) (*n*,%)	OR(95% CI)	*P* value
Maternal age (years)				0.040
18–39	207(98.6)	3080(95.4)	3.33(1.05–11.1)	
≥40^a^	3(1.4)	149(4.6)		
Ethnic				0.048
Han-Zhuang^a^	190(90.5)	3033(93.9)		
Minority	20(9.5)	196(6.1)	1.63(1.01–2.64)	
Education level				
≤ 9 years of school^a^	91(43.3)	1422(44.0)		
10–12 years of school	67(31.9)	1116(34.6)	0.94(0.68–1.30)	0.700
≥ 13 years of school	52(24.8)	69(21.4)	1.18(0.83–1.67)	
Current smoking				
No^a^	209(99.5)	3195(98.9)		
Yes	1(0.5)	34(1.1)	0.45(0.06–3.30)	0.432
Previous miscarriage				
No^a^	195(92.9)	2942(91.1)		
Yes	15(7.1)	287(8.9)	0.79(0.46–1.35)	0.388
Pregnancy-induced hypertension				
No^a^	197(93.8)	3078(95.3)		
Yes	13(6.2)	151(4.7)	1.35(0.75–2.41)	0.320
Gestational diabetes				
No^a^	159(75.7)	2397(74.2)		
Yes	51(24.3)	832(25.8)	0.92(0.67–1.28)	0.634
Parity				
1-2^a^	205(97.6)	3088(95.6)		
≥ 3	5(2.4)	141(4.4)	0.53(0.22–1.32)	0.174
Maternal previous antibiotic use				
No^a^	208(99.0)	3131(97.0)		
Yes	2(1.0)	98(3.0)	0.31(0.07–1.26)	0.100

*OR* odds ratio, *95%CI* 95% confidence interval

^a^indicates the reference group in the calculation of OR

**Table 2:** Multivariate logistic regression analysis of risk factors associated with GBS colonization among pregnant women

Variables	OR	95% CI	*p* value
Maternal age (≥40 yrs. vs. < 40 yrs^a^)	0.30	0.09–0.95	0.040
Ethnicity (minority vs. Han-Zhuang^a^)	1.62	1.00–2.63	0.049

*OR* odds ratio, *95%CI* 95% confidence interval

^a^indicates the reference group in the calculation of OR

### Risk factors and outcomes for infants GBS colonization

Of the 3439 infants in the study, 377 (10.9%) were born prematurely (< 37 weeks’ gestation). There were two cases of culture-proven GBS positive EOD: one from blood and the other from the cerebrospinal fluid. Four other cases were positive for other pathogens (two were *Escherichia coli*, one was *Candida albicans* and the other was *Staphylococcus aureus*) from blood cultures. One infant died during delivery with GBS infection revealed by growth of the pathogen from a blood culture obtained upon delivery. The EOD incidence from GBS was 0.58 per 1000 live births while the infant mortality rate of GBS was 0.29 per 1000 live births.

Twenty-three of the 3439 neonatal samples were positive for growth of GBS, with an estimated colonization rate of 0.7%. Among the 210 mothers who were positive for GBS by culture, only 16 of their infants were GBS positive, resulting in a 7.6% vertical transmission rate.

Table 3 shows the association between characteristics of mothers at labor and infant GBS colonization in univariate analysis. GBS colonization in mother (*p* < 0.001), episiotomy (*p* = 0.020), vaginal birth (*p* = 0.027), longer duration of membrane rupture (≥12 h) (*p* < 0.001), and longer labor (≥4 h) (*p <* 0.001) were all significantly associated with GBS colonization in newborns. In multivariate analysis, only maternal GBS colonization (*p* < 0.001), duration of membrane rupture ≥18 h (*p* < 0.001) and length of labor ≥4 h (*p <* 0.001) were included in the final model predicting risk of GBS colonization in infants (Table 4).

**Table 3:** Association between maternal characteristics and potential risk factors for neonatal GBS colonization

Variables	GBS colonization (*n* = 23) (*n*,%)	GBS negative (*n* = 3416) (*n*,%)	OR(95%CI)	*P* value
Maternal age (years)				
18–39	23(100.0)	3264(95.6)	0.00(0.00–0.00)	0.996
≥40^a^	0(0.0)	152(4.4)		
Ethnic				
Han-Zhuang^a^	22(95.7)	3201(93.7)		
Minority	1(4.3)	215(6.3)	0.68(0.09–5.05)	0.703
Education level				
≤ 9 years of school^a^	9(39.1)	1504(44.0)		
10–12 years of school	10(43.5)	1173(34.3)	1.43(0.58–3.52)	0.443
≥ 13 years of school	4(17.4)	739(21.6)	0.91(0.28–2.95)	0.868
Current smoking				
No^a^	23(100.0)	3381(99.0)		
Yes	0(0.0)	35(1.0)	0.00(0.0–0.0)	0.998
Previous miscarriage				
No^a^	23(100.0)	3114(91.2)		
Yes	0(0.0)	302(8.8)	0.00(0.0–0.0)	0.998
Pregnancy-induced hypertension				
No^a^	31(91.3)	3254(95.3)		
Yes	2(8.7)	162(4.7)	1.91(0.45–8.23)	0.384
Gestational diabetes				
No^a^	20(87.0)	2536(74.2)		
Yes	3(13.0)	880(25.8)	0.43(0.13–1.46)	0.176
Parity				
1-2^a^	23(100.0)	3270(95.7)		
≥ 3	0(0.0)	146(4.3)	0.00(0.0–0.0)	0.996
Cesarean section				
No^a^	18(78.3)	1844(54.0)		
Yes	5(21.7)	1572(46.0)	0.33(0.12–0.88)	0.027
Episiotomy				
No^a^	14(60.9)	2764(80.9)		
Yes	9(39.1)	652(19.1)	2.73(1.17–6.32)	0.020
Membrane rupture				
< 11 hours^a^	15(65.2)	3376(98.8)		
≥ 12 h	8(34.8)	40(1.2)	45.01(18.06–112.16)	< 0.001
Length of labor				
< 4 hours^a^	11(47.8)	3165(92.7)		
≥ 4 hours	12(52.2)	251(7.3)	13.75(6.01–31.48)	< 0.001
Maternal previous antibiotic use				
No^a^	23(100.0)	3316(97.1)		
Yes	0(0.0)	100(2.9)	0.00(0.0–0.0)	0.997
Gestational age				
Full-term≥37weeks^a^	21(91.3)	3041(89.0)		
Preterm< 37 weeks	2(8.7)	375(11.0)	0.77(0.18–3.31)	0.728
Maternal GBS colonization				
No^a^	7(30.4)	3222(94.3)		
Yes	16(69.6)	194(5.7)	37.96(15.44–93.36)	< 0.001

*OR* odds ratio, *95%CI* 95% confidence interval

^a^indicates the reference group in the calculation of OR

**Table 4:** Multivariate logistic regression analysis of risk factors associated with GBS colonization among infants

Variables	OR	95% CI	*P* value
Membrane rupture (≥12 h vs. < 12 hrs^a^)	125.75	30.72–514.76	< 0.001
Length of labor (≥4 h vs. <4hrs^a^)	10.64	3.16–35.86	< 0.001
Maternal GBS colonization (yes vs. no^a^)	21.52	7.03–65.85	0.001

*OR* odds ratio, *95%CI* 95% confidence interval

^a^indicates the reference group in the calculation of OR

Infants colonized with GBS were more likely to have had a temperature of ≥38 °C (*p* < 0.001), developed EOD (*p* < 0.001), and received antibiotics (*p* < 0.001) (Table 5). Infants with GBS were also more likely to have been admitted to the NICU (*p* < 0.001) with a longer hospital LOS (11 vs. 3 days; *p* < 0.001).

**Table 5:** Neonatal GBS colonization outcomes

Variables	GBS colonization (*n* = 23) (n,%)	GBS negative (*n* = 3416) (n,%)	*P* value
Gender			0.122
Male	16(69.6)	1813(53.1)	
Female	7(30.4)	1603(46.9)	
Birth weight, gram	3126 ± 52	2895 ± 55	0.036
Neonatal fever			< 0.001
No	20(87.0)	3412(99.9)	
Yes	3(13.0)	4(0.1)	
Neonatal antibiotic use			< 0.001
No	19(82.6)	3412(99.9)	
Yes	4(17.4)	4(0.1)	
Transferred to NICU			< 0.001
No	15(65.2)	3413(99.9)	
Yes	8(34.8)	3(0.1)	
Length of stay (days) Mean (range)	10.9(4–34)	3.1(2–18)	< 0.001
Occurrence of EOD			< 0.001
No	21(91.3)	3412(99.9)	
Yes	2(8.7)	4(0.1)	

*NICU* neonatal intensive care unit; *EOD* early onset diseases

## Discussion

Our study demonstrates that the GBS colonization rate in pregnant Chinese women in Liuzhou was 6.1%, which is similar to the reported rate of 7.1% in Beijing [9] and 8.2% in Dongguan [10]. Our rate is similar to those of other Asian countries including Philippines (7.5%) [11], Japan (8.2%) [12], Myanmar (7.1%) [11] and South Korea (8.0%) [13]. but lower than the global colonization rate of 17.9% reported by a recent meta-analysis study [14]. The GBS colonization rate in our study was also lower than the rate reported in Southeast Asia (11.1%) [14]. These results indicate that GBS colonization prevalence in pregnant women varies widely across the globe. Factors that may have contributed to this variation include the level of economic development, the availability of medical care and preventive services, varying clinical practice guidelines, as well as differing average child bearing age [15]. Our study found that ethnic minority pregnant women in Liuzhou were significantly more likely to be colonized with GBS when compared with Han and Zhuang ethnic groups. This is consistent with the previous study conducted in US which showed higher GBS colonization rates were more likely to have black and Hispanic maternal races than white race [16–19].

In the present study, we found that there was a significant association between maternal GBS colonization and newborn GBS colonization. Studies have found that the risk factors for infant GBS colonization include maternal rectovaginal colonization, preterm delivery, low birth weight, antepartum and intrapartum fever and prolonged membrane rupture [20–22]. Although we found no association between maternal GBS colonization and preterm birth, we did find the association between both membrane rupture (≥12 h) duration and labor duration (≥4 h) with infant GBS colonization risk. Our data was consistent with a previous study conducted in China which showed that prolonged rupture of membranes and increased duration of rupture of membranes were significant risk factors for neonatal GBS colonization [23].

Our data show that vertical transmission from mothers to their newborns was 7.6%. This is lower than what was reported in Europe and Asia, which have a transmission rate of 11.2% in Germany [24], 15.1% in Bangladesh [25] and 16.7% in Taiwan [26]. It is known that birth canal contact is a risk factor of GBS transmission to newborns [27]. The cesarean section rate at LMCHH was 54.1%, while the cesarean section rate was 42% in mainland China [28]. The high cesarean section rate may explain in part the low rate of vertical transmission in our study. It is also worth noting that despite the implementation of a universal protocol of GBS screening procedures in our institution, we still observed 7 infants with GBS colonization who were born to GBS-negative mothers. It was reported that false negative results are expected to occur based on several procedure of the implementation of screening test for GBS [16]. Improper collection methods, delay in processing, insufficient laboratory techniques, recent antibiotic use, or delay in GBS colonization may all have contributed to the negative GBS screening results in mothers who deliver GBS positive infants [18, 29, 30]. Several studies have examined the risk of GBS colonization in infants who were born to GBS negative mothers. The reported rates by these studies, ranged between 21.5 and 61.4%, [16, 31, 32] were consistent with our study observed rate of 30.4% (7/23). To improve the accuracy of screening by identifying the GBS status, a rapid polymerase chain reaction (PCR) test could be used at the time of labor to increase the sensitivity and specificity of the tests [33–35].

Our data demonstrated that GBS colonization remains an important cause of infant morbidity requiring NICU admission and longer LOS. Clinically, infants may be colonized with GBS through intra-amniotic infection by way of GBS ascending from the vagina or through birth canal contact during labor [27]. Exposure to GBS increased the risk of GBS-EOD, which often leads to NICU admission [26]. A previous study indicated that infants born to GBS positive mothers were at a higher risk of admission to the NICU [36], which is consistent with our study findings. Possible reasons for the increased morbidity of GBS positive infants include exposure to GBS in the perinatal period [27], hypervirulence of certain GBS strains [37], as well as the “maternal antibody” hypothesis [38]. The serotype III of GBS is considered hypervirulent which may cause a longer colonization duration throughout the pregnancy and is the most common serotype isolated in neonates less than 7 days of age [21]. In addition, maternal serum IgG antibodies against specific capsular polysaccharides of GBS has been shown to be protective against infection with virulence strain of GBS in their offspring [31]. Mothers with low titers of IgG antibodies against the GBS strains may be responsible for the hypervirulence of GBS [38], thus contributing to the GBS colonization and possible future infection in their newborns.

This study possessed two strengths. This is the first large-scale study conducted in western China with a cohort consisting of 3439 pairs of mothers and infants. Additionally, we collected surface culture on all newborns regardless of maternal GBS culture results.

There are some limitations to our study. First of all, the single-center design and the low prevalence of GBS positive infants may limit the generalizability of our study results. Secondly, the incidence of GBS-EOD may have been underestimated because we did not follow the infants who were discharged from hospital before 7 days. Lastly, due to budget constraints, the study utilized a culture-based testing procedure which is less expensive but not as sensitive as the PCR test.

## Conclusions

Our study revealed that maternal age as well as ethnicity may play a role in maternal colonization of GBS. Maternal colonization with GBS, longer duration of membrane rupture and labor were significant risk factors for GBS colonization in Chinese newborns. Outcomes of infants colonized with GBS included fever, increased need for antibiotic, NICU admission as well as longer LOS. Future studies are needed to establish guidelines for GBS screening and Intrapartum Antibiotic Prophylaxis use in China. Prospective studies are also needed to evaluate the GBS disease burden in Chinese mothers and infants nationwide.

## Abbreviations

95%CI: 95% confidence interval
EOD: Early onset diseases
GBS: *Group b streptococcus*
IAP: Intrapartum antibiotic prophylaxis
NICU: Neonatal intensive care unit
OR: Odds ratio
